# The relationship between ethical climate and organizational cynicism: mediating role of match and identification with the organization

**DOI:** 10.1038/s41598-025-97415-3

**Published:** 2025-04-09

**Authors:** Marcin Wnuk, Marta Żywiołek-Szeja, Agata Chudzicka-Czupała

**Affiliations:** 1 Department of Work and Organizational Psychology, Adam Mickiewicz University in Poznań, ul. Szamarzewskiego 89/AB, Poznań, 60 – 568 Poland; 2 Faculty of Psychology in Katowice, Interdisciplinary Center for Social Activity & Well-Being Research FEEL & ACT WELL, SWPS University, Katowice, Poland

**Keywords:** Human behaviour, Occupational health, Psychology

## Abstract

Depending on its type, the ethical climate of an organization has either beneficial or detrimental effects in the workplace. However, there is a lack of knowledge about the role of employees’ attachment to the organization and the coherency of values between employees and organizations in the relationship between different types of ethical climates and negative attitudes toward the organization. This cross-sectional study tested the mechanisms of the direct and indirect effects of ethical climate on organizational cynicism through person–organization fit, organizational pride, and affective commitment, attempting to determine the protective function of different ethical climates on organizational cynicism, which is an undesirable workplace phenomenon. The study focused on 1071 Polish employees from different business areas with contracts of employment. The results showed that an instrumental ethical climate had positive direct and indirect effects on organizational cynicism through all three mediators, person–organization fit, organizational pride, and affective commitment. Moreover, beneficial indirect effects of caring and independent climates on organizational cynicism through person–organization fit, organizational pride, and affective commitment were also confirmed. Law and code and rules climates were positively and indirectly related to organizational cynicism via organizational pride but not organizational affective commitment. Discussed the impact of different ethical climates on organizational cynicism and the beneficial role of person-organization fit, organizational pride, and affective commitment underlying this relationship, emphasizing the preventing function of organizational attachment, organizational pride, and values consistency between employee and organization in referring cynical attitudes in the workplace.

## Introduction

Organizational cynicism is an undesirable phenomenon in the workplace, leading to negative outcomes, such as reduced performance^1,2^, work engagement^3^ and increased turnover intentions^4,5^. A cynical attitude at work is rooted in a lack of trust in the organization and its agents^6^. Despite the progress in the trust index in the last few years, Poles are still one of the most distrustful nations, which also applies to trustful in business^7,8^. According to Edelman’s trust barometer in 2017, Poland was placed in the last position regarding trust in institutions and also significantly lower than the world average in reference to trust in employers^7^. The 2022 social survey data indicated that 29% of Poles declared trust in strangers and 34% trust in business partners^8^.

It is challenging for company representatives to determine how to increase the level of trust in organizations, employees’ belongingness, and affective attachment to protect against detrimental attitudes in the workplace, such as organizational cynicism^6^. A promising area of inquiry is the organizational ethical climate^9^, which is a predictor of employees’ attitudes at work^10,11^ and can be tested as a potential protecting factor from cynical behaviors in the workplace. However, this variable has not been explored as a potential antecedent of cynical attitudes in the workplace despite both of these constructs referring to ethical values, rules, norms, and behaviors in the workplace^6,10,11^. This study is the first attempt to fill this gap by verifying the potential mechanisms underpinning the link between ethical climate and organizational cynicism with three mediators: person-organization fit^12^, organizational pride^13^, and affective commitment^14^. Although they are three different constructs, they have one common core element: positive identification with the organization and satisfaction with the need for belongingness. For employees, the congruency of their values with the values of organizations makes it a more attractive and desirable workplace, which has consequences on their job satisfaction^15^. Also, an emotional bond with the organization and pride from being an organizational member results in a positive attitude toward the organization^13,16,17^.

Based on the above and results of previous studies^4,18,19^ I think that employees who fit into the organization, have an emotional bond with the organization, and are proud of will not manifest organizational cynicism.

On the other hand, the ethical norms, rules, and values included in an organization’s ethical code of conduct can be the attractive element with whom employees want to identify, be proud because of that, and build an emotional bond with the organization. Earlier research indicated ethical climate as a predictor of organizational affective commitment^10,11^.

These results suggest that an ethical climate is the antecedent of fit between employees and the organization, as well as organizational pride and emotional attachment to the organization. This study aims to examine the direct and indirect effects of ethical climate on organizational cynicism through the person-organization fit, organizational pride, and organizational affective commitment.

## Ethical climate and its consequences

An ethical climate is conceptualized as a “prevailing perception of typical organizational practices and procedures that have ethical content.”^9^ The theoretical grounds of the ethical organizational climate are the ethical philosophy dimensions (egoism, benevolence, and principled) and three levels of decision-making (individual, local, and cosmopolitan). Every type of climate consists of one ethical philosophy component, which influences one of the levels of decision-making^9^. Combinations of these two elements result in nine types of ethical climates, among which five have been empirically confirmed: caring, rules, law and codes, instrumental, and independence^10^. For example, a caring climate adopts the benevolence ethical philosophy approach and refers to the individual level of decision-making, which leads to friendship as a main value in the organization. Contrary to caring, an instrumental climate is based on egoism as an ethical criterion and regards the individual dimension of decision-making, which makes the central value in the organization become self-interest^9^.

In a caring climate, the most desirable state in the organization is the well-being of the largest possible group of stakeholders, and altruistic behaviors are considered precious and highly valued^9^. Conversely, the instrumental climate focuses on individual interests and desires, encouraging and rewarding expressions of selfishness in goal achievement^9^.

In the rules climate, decisions are made in accordance with the organizational politics, procedures, and codes of conduct, which are characteristic and specific to the organization and its autonomous moral values^9^. In an independent climate, decisiveness is based on employees’ points of view, their values and beliefs, and a moral codex without any reference to external norms or standards. In a law and code climate, the ethical organizational signposts are based on external, objectified standards, such as legal norms or the Bible^10^.

Previous studies have indicated that unlike an instrumental climate, which leads to detrimental consequences, the other types of ethical climates are profitable or irrelevant both for organizations and employees^10,11^. For example Wang and Hsieh^20^ found that an instrumental climate was negatively related to job satisfaction, while the other types of ethical climates were positively correlated with job satisfaction. In contrast, Tsai and Huang^21^ found law and code climate was the only type of ethical climate that was not significantly associated with job satisfaction.

Victor and Cullen’s^9^ ethical climate has never been examined with organizational pride and person–organization fit. Based on a longitudinal study, Halbusi et al.^22^ reported that the beneficial function of person–organization fit strengthened the positive relationship between the ethical climate and employees’ ethical behavior. In Lopez et al.’s^23^ study, ethical work climate dimensions were found to be correlated differently with person–organization fit among retail employees in Japan and the US.

Moreover, ethical climate manifestations have never been tested as an antecedent of organizational cynicism, although studies have highlighted its role in predicted ethical and unethical intentions and behaviors like bullying^24^, altruism, identifying with the company and protecting company resources^25^, stealing, lying, and obeying^26^ as well as emphatic concerns, personal morality, kindness, and common humanity^27^. Due to the different ethical climate types used in these studies, however, their findings are not conclusive. Additionally, the vast majority of analyses have been limited to examining simple correlation effects without considering the potential mechanism of influence^10,11^. For example, in Leung’s research, only the perception of instrumental and caring climates was irrelevant to altruism^25^.

This study attempts to fill this gap in the literature, positioning ethical climate manifestations as direct and indirect antecedents of organizational cynicism.

### Organizational cynicism antecedents

Organizational cynicism is an attitude characterized by negative perceptions, emotions, and behaviors focused on the organization^28^. Cognitive aspects of organizational cynicism include perceiving an organization as two-faced, inconsistent, dishonest, devoid of any sense of justice, incongruous in its declarations and activities, indifferent to the well-being and needs of employees while feigning interest, and focused only on its own selfish interests. In employees, this can elicit reactions like disappointment, frustration, disorientation, aversion, helplessness, the feeling of being cheated, and a desire for revenge^28^. Ultimately, it leads to employee reciprocal negative reaction, resulting in a plethora of non-loyal behaviors directed against the organization, such as criticism, unfavorable verbal and non-verbal behavior, negative interpretations of organizational activities, and cynical predictions about the organization’s action in the future^28^.

Some studies have emphasized the buffering role of certain factors in organizational cynicism, such as organizational justice, perceived organizational support, or positive affectivity^5,6^. For example, Cicek et al.^5^ found that the employees perception of an organization as providing support prevented cognitive, affective, and behavioral expressions of cynicism.

Only one study considered the link between organizational cynicism and organizational pride, finding a negative relationship between most aspects of both constructs^18^. Further, affective commitment has not been explored as a protective factor against organizational cynicism. However, the promising findings reported by Lapointe et al.^19^ indicate that all commitment forms are negative antecedents of cynical attitudes at work. Organizational pride is a distinct but similar construct to affective commitment, which has a broader range and captures aspects of pride. Both concepts reflect a favorable attitude toward the organization but are based on different premises and manifest differently. Affective commitment is grounded in belongingness and reflects reciprocity and a bond with the organization^29^, whereas pride is an emotional state that results from employees’ perceptions of the values and achievements of the organization^18^.

Person–organization fit is a part of a more global construct and reflects complementary or supplementary mutual matching between an employee and the organization^30^, which leads to many positive outcomes for both parties^12^. Only one study has considered the role of person organization–fit in organizational cynicism, confirming that perceived value similarity can reduce employees’ intention to quit due to a reduction in cynicism^4^.

## Person–organization fit, organizational pride, and affective commitment as mediators

Most studies have regarded the ethical climate as a mediator or moderator^11^ without considering it as a dependent variable, which, by establishing expectations about morally appropriate attitudes with which employees can identify, encourages employees to behave in congruence with organizational ethical standards.

The framework of the present study is grounded in situational strength theory^31^ and social identity theory^32^. Situational strength is defined “as implicit or explicit cues provided by external entities regarding the desirability of potential behaviors.” Due to the perception of an ethical climate, which is both an environmental and contextual factor, employees feel obligated to act according to the signals and directions provided, viewing them as behavioral standards. This psychological pressure arising from the organizational environment is stronger when the cues that come from it are more consistent and clearer, when the consequences of an employee’s decisions or actions are more serious, and when constraints on the employee’s decision-making are beyond the employee’s control^31^. These suggestions result from the perception of the ethical climate as a point of reference for decision-making and differ depending on the source of the ethical content of organizational politics and procedures and the values and code of conduct they entail. They also have informative functions, showing what ethical values and rules are significant and desirable for organizations, what behaviors are expected and will be strengthened, and what is undesirable and will be stigmatized and excluded.

For instance, in a caring climate, the main values are altruism, helping, providing support, and cooperation to guarantee the well-being of as many employees as possible. Conversely, in an instrumental climate, selfish and competitive behaviors are expected and rewarded as an expression of employees’ pursuit of individual well-being. Drawing on social identity theory^32^, it is possible to compare the organizational values and ethics with those possessed by employees and, in line with person–organization fit theory^30^, mutual matching based on strengthened organizational identification protects against harmful behaviors in the workplace like cynicism^4^.

According to social identity theory, affective organizational commitment acts as a mediator in the connection between ethical climate connection and organizational cynicism. Previous studies have shown that the organizational ethical climate predicts affective organizational commitment^33^. Employees perceive the ethical climate not only by becoming familiar with procedures, policies, and standards of conduct but also by being rewarded for attitudes that are congruent with organizational cues and imperatives and punished and discouraged from ones that are incongruent. Moreover, employees’ perception of the organizational ethical climate, values, and rules—and the favorable treatment they receive for respecting them—can build an affective attachment to the organization based on a feeling of belongingness and a sense of purpose in organizational membership. In turn, this attitude can prevent cynical behaviors and promote positive ones, for example, enhancing employees’ intention to stay at the organization^34^. Indeed, Teresi et al.^35^ found that an ethical climate of friendship, through stronger organizational identification, is related to stronger organizational commitment, making employees less willing to leave the organization.

The third mechanism through which organizational pride mediates the link between organizational climate and cynicism is connected with employees’ perception of the attractiveness of the organization’s moral values, leading to pride in the organization and reducing cynical attitudes. However, there is a paucity of studies regarding the antecedents of organizational pride, and most have been limited to considering corporate social responsibility as a premise of organizational pride^36–38^. The ethical climate is promising concept for organizational pride. Ethical values are important for organizations, and sharing them with employees through the social identification process^32^ can amplify employees’ psychological bond with the organization^39^, enhancing their self-image and self-worth^30^. For employees, the ethical standards of an organization can be the point of reference for evaluating their organizational status (pride) and, accordingly, improving their own status as organizational members. This allows them to realize their ethical aspirations by modeling organizational ethics standards.

Sturm et al.^40^ argued that organizational virtuousness fosters organizational pride, and through this variable it is indirectly positively related to task performance and citizenship behaviors toward the organization. Other beneficial behaviors are also tied to the feeling of pride in the organization, such as reduced turnover intentions^41,42^ and customer-oriented behavior^43^, which suggests that pride could help to prevent organizational cynicism.

## Present study

**Fig. 1:**
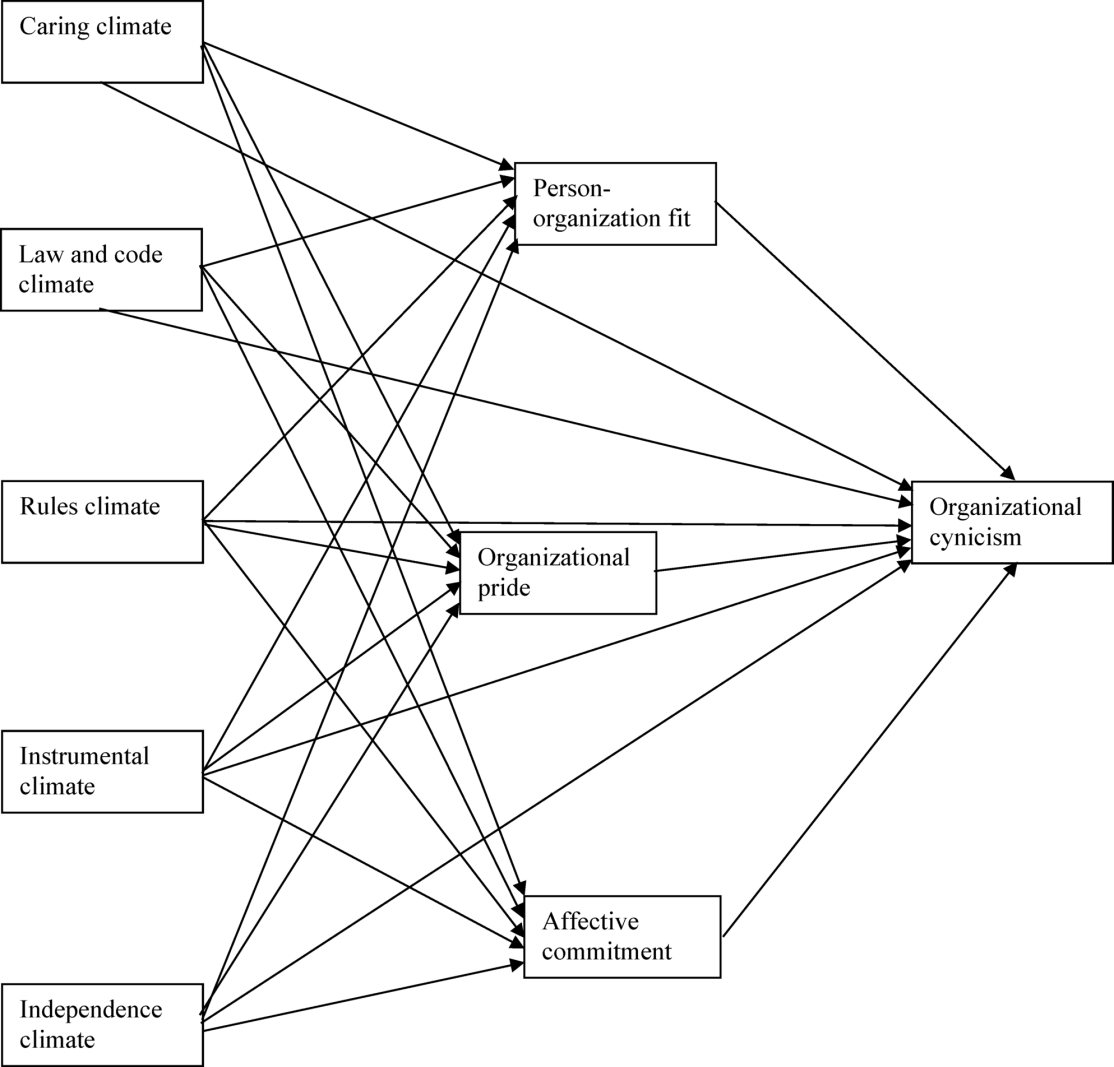
Conceptual model.

Based on the considerations presented above, a positive relationship was anticipated between the types of ethical climate, except for the instrumental climate, person–organization fit, organizational pride, and affective commitment (Hypothesis 1). Meanwhile, a negative relationship was expected between these three variables and organizational cynicism (Hypothesis 2). Additionally, negative direct and indirect effects of the different ethical climate types were expected, except for the instrumental climate and organizational cynicism through person–organization fit, organizational pride, and affective commitment (Hypothesis 3). In line with this hypothesis, it was assumed that instrumental climate is directly and indirectly positively related to organizational cynicism. All hypotheses are summarized within the conceptual model presented in Fig. 1.

## Method

### Study design and participants

The study was cross-sectional and employed a convenience sampling method. The criteria for participation were adulthood and actively working as an employee. The study was conducted in accordance with the Declaration of Helsinki and received approval from the Ethics Committee of the Department of Psychology at University of Social Sciences and Humanities (SWPS) University (No. WKEB89/11/2023). All participants provided written informed consent to participate in the study.

### Setting

Participants were recruited from an SWPS University database as well as with the snowball method. Adulthood and corporate employment were the main inclusion criteria. Data were collected through the online platform.

### Participants

The study sample consisted of 1071 participants. Most were women (860, 80.3%), 200 were men (18.7%), and 11 reported another sex (1%). The average age of the participants was M = 28.64 years (SD = 8.74), with average seniority of M = 7.88 years (SD = 7.04). Two (0.2%) of the participants had elementary education, four (0.4%) had vocational education, 539 (50.3%) had secondary education, and 526 (49.1%) had higher education. With respect to the position held, 432 (40.3%) were ordinary workers, 412 (38.5%) were independent specialists, 50 (4.7%) were low-level managers, 106 (9.9%) were middle-level managers, and 71 (6.6%) were senior-level managers. In terms of company size, 278 participants (26%) were working in micro-organizations, 254 (23.7%) in small companies, 152 (14.2) in medium-sized companies, and 387 (36.1%) in large companies.

### Variables

The independent variables were types of organizational ethical climate, organizational cynicism was the dependent variable, and three potential mediators in these relationships were examined: person-organization fit, organizational pride, and affective commitment. The models were controlled by sex, age, education, seniority, position held in the organization, and company size. Additionally, in every tested model in which an ethical climate type served as an independent variable, the other ethical climates were introduced as controlled variables.

### Measurement

#### Ethical work climate questionnaire

The ethical work climate was verified using the Polish version of the Ethical Work Climate Questionnaire (EWCQ)^44^. This tool examines employees’ perceptions of the five types of ethical work climate: instrumental, caring, law and code, rules-oriented, and independence^9^. The Polish version of the EWCQ consists of 20 items. Every dimension of the EWCQ includes four items as indicators. Items are rated on a five-point Likert scale (from 1 = strongly disagree to 5 = strongly agree). This tool has been shown to have satisfactory internal consistency^9^. The CFA confirmed five factors of EWCQ (χ2[1] = 3.36; *p* = .064; CMIN/df = 1.2; GFI = 0.99; CFI = 0.99; NFI = 0.99; TLI = 0.99; RMSEA = 0.022 (90% CI [0.000.033]); SRMR = 0.043). The five-factor solution explained 70.98% of the ethical organizational climate, 31.75% of the law and code ethical organizational climate, 17.23% of the independence ethical organizational climate, 10.88% of the caring ethical organizational climate, 6.56% of the rules ethical organizational climate, and 4.5% of the instrumental ethical organizational climate.

#### Organizational cynicism questionnaire

Organizational cynicism was measured using Organizational Cynicism Questionnaire^45^. This tool consists of nine items, which are combinations of six items from the Organizational Cynicism Scale^46^ and three from a tool prepared by Eaton and Struthers^47^. The one-factor structure of this measure has demonstrated good reliability based on the Cronbach alpha coefficient^45^. Participants responded on a five-point Likert scale ranging from 1 = strongly disagree to 5 = strongly agree. In this study, this one-factor solution explained 69.66% of the variance in organizational cynicism, supported by the results of the CFA (χ2[18] = 57.21; *p* = .000; CMIN/df = 3.17; GFI = 0.98; CFI = 0.99; NFI = 0.99; TLI = 0.99; RMSEA = 0.045 (90% CI [0.032.059]); SRMR = 0.011).

#### Organizational pride scale

The Polish version of Organizational Pride Scale^42,48^ was used to verify organizational pride. It consists of seven items in the form of a one-dimensional scale. The answers are given on a five-point Likert and range from 1 = strongly disagree to 5 = strongly agree. In this study, the one-factor solution was confirmed as appropriate. In this study, CFA showed that the one-factor solution fit well (χ2[5] = 11.50; *p* = .042; CMIN/df = 2.30; GFI = 0.99; CFI = 0.99; NFI = 0.99; TLI = 0.99; RMSEA = 0.035 (90% CI [0.006.062]); SRMR = 0.006), explaining 70.65% of the variance in organizational pride.

#### Perceived person-organization fit

Person–organization fit was measured by the Polish version of Perceived Person-Organization Fit tool^13,49^. This tool consists of three items, with responses rated on a five-point Likert scale ranging from 1 = strongly disagree to 5 = strongly agree. In this study, the CFA showed that the three-item solution fit the data well (χ2[1] = 3.36; *p* = .067; CMIN/df = 3.36; GFI = 0.99; CFI = 0.99; NFI = 0.99; TLI = 0.99; RMSEA = 0.047 (90% CI [0.000.106]); SRMR = 0.011), explaining 85.52% of the variance in this construct.

#### Organizational commitment scale

Affective organizational commitment was examined using the Polish version of the Organizational Commitment Scale^50,51^. This measure has good psychometrical properties^51^. Responses were rated on a five-point Likert scale ranging from 1 = strongly disagree to 5 = strongly agree. Four out of six indicators of affective organizational commitment were chosen. The CFA confirmed the fit of the four-item solution (χ2[1] = 0.86; *p* = .355; CMIN/df = 0.86; GFI = 1; CFI = 1; NFI = 1; TLI = 1; RMSEA = 0.000 (90% CI [0.000.079]); SRMR = 0.003), which explained 55.21% of the variance in affective organizational commitment.

### Study size

To assess whether the sample size was large enough, a power analysis was applied, using G*Power 3.1.9.7. with linear multiple regression, a fixed model, and R quadrant deviation from 0. A medium effect size of 0.17 with an alpha of 0.05, a power of 0.95, and 14 predictors were chosen. The results showed that 194 participants was the required sample size, which was less than 20% of the research sample.

### Statistical methods

Statistical analyses were performed using the IBM SPSS and IBM SPSS statistical AMOS packages version 26. Before models with mediators were examined, the data were tested for normal distribution and potential multicollinearity problems. The data distribution is close to normal when the skewness is within the interval of − 2 and 2 and the kurtosis is between − 10 and 10^52^. Multicollinearity was verified using the variance inflation factor (VIF), which did not exceed 5^53^.

Additionally, to assess the discriminant validity of the research measures, a CFA was performed using AMOS based on the following fitness indices: the model’s χ^2^, the minimum discrepancy/degrees of freedom (χ^2^/*df*), the comparative fit index (CFI), goodness-of-fit index (GFI), Tucker–Lewis index (TLI), normed fit index (NFI), incremental fit index (IFI), root-mean-square error of approximation (RMSEA), and standardized root-mean residual (SRMR). The results indicated a good model fit (χ^2^ > 0.05; χ^2^/*df* < 3; CFI ≥ 0.95; GFI, TLI and NFI, all ≥ 0.90; and RMSEA and SRMR < 0.08)^54–56^. In addition to the Cronbach alpha coefficient, the convergent validity of the research tools was examined using the composite reliability method. According to Bagozzi and Heatherton^57^, the composite reliability value should exceed 0.70, the magnitude of most factor loadings should be more than 0.50, and the average variance extracted should exceed 50.

The indirect effects assumed in the hypotheses were computed using model 4 of the SPSS macro PROCESS, applying the bootstrapping method^58^. The 95% confidence interval (CI) was calculated based on 5,000 bootstrapped replications. A coefficient is deemed statistically significant if it does not include 0 in its 95% CI.

### Descriptives and preliminary results

The highest VIF in the dataset results, which slightly exceeded 2, suggested a lack of multicollinearity (see Table 1). Further, the range of skewness and kurtosis values fell in the interval (–1;1), indicating that the distribution of the variables did not significantly differ from the normal distribution, which was the premise for using the maximum likelihood method in the CFA (see Table 1). The results of the CFA showed that only the nine-factor solution was a good fit for the data (Table 2). Following Bagozzi and Heatherton’s^57^ criteria, the research measures showed good convergent validity (see Table 3). The composite reliability values were much higher than the required 0.70. The magnitude of all factor loadings was greater than 0.50, and the average variance extracted was greater than 50%, with the only exception being instrumental climate.

**Table 1:** Descriptive statistics (*N* = 1071).

Variable	Minimum	Maximum	Mean	Standard deviation	Skewness	Kurtosis	VIM	Cronbach’s alpha coefficient	CR
Caring climate	4	20	12.30	3.80	− 0.32	− 0.27	1.98	0.87	0.81
Law and code climate	4	20	13.31	3.61	− 0.29	− 0.23	2.02	0.86	0.84
Rules climate	4	20	14.31	3.63	− 0.59	0.27	2.06	0.86	0.82
Instrumental climate	4	20	11.15	3.58	0.12	− 0.29	1.26	0.79	0.79
Independence climate	4	20	11.10	3.64	− 0.15	− 0.35	1.39	0.86	0.84
Person-organization fit	3	15	13.87	3.30	− 0.27	− 0.55	2.16	0.91	0.88
Organizational pride	7	35	24.56	6.81	− 0.54	− 0.19	2.21	0.93	0.93
Affective organizational commitment	4	20	12.46	4.50	− 0.19	− 0.78	1.96	0.87	0.92
Organizational cynicism	9	45	22.82	9,89	0.37	− 0.82		0.94	0.94

*VIM*  variance inflation factor, *CR* component reliability.

**Table 2:** The results of CFA for all measures used in the research measures (*N* = 1071).

Number of model	Factors	CMIN/DF	RMSEA	SRMR	GFI	CFI	TLI	NFI
One factor model	IC, CC, RC, LC, IDC, OP, AC, PO, C	11.59 (*p* = .000)	[0.099, 90% (0.098; 0.101)]	0.093	0.67	0.77	0.72	0.75
Two factor model	IC + CC, RC, LC, IDC, OP, AC, PO, C	11.12 (*p* = .000)	[0.097, 90% (0.095; 0.099)]	0.091	0.68	0.78	0.73	0.76
Three factor model	IC + CC + RC, LC, IDC, OP, AC, PO, C	11.05 (*p* = .000)	[0.097, 90% (0.095; 0.099)]	0.091	0.68	0.78	0.73	0.76
Four factor model	IC + CC + RC + LC, IDC, OP, AC, PO, C	10.09 (*p* = .000)	[0.092, 90% (0.090; 0.094)]	0.086	0.69	0.80	0.76	0.78
Five factor model	IC + CC + RC + LC + IDC, OP, AC, PO, C	8.22 (*p* = .000)	[0.082, 90% (0.080; 0.088)]	0.070	0.73	0.84	0.81	0.82
Six factor model	IC + CC + RC + LC + IDC + OP, AC, PO, C	7.08 (*p* = .000)	[0.077, 90% (0.074; 0.077)]	0.064	0.76	0.87	0.84	0.85
Seven factor model	IC + CC + RC + LC + IDC + OP + AC, PO, C	6.88 (*p* = .000)	[0.074, 90% (0.072; 0.076)]	0.063	0.76	0.87	0.84	0.85
Eight factor model	IC + CC + RC + LC + IDC + OP + AC + PO, C	4.70 (*p* = .000)	[0.059, 90% (0.057; 0.061)]	0.065	0.84	0.92	0.90	0.90
Nine factor model	IC + CC + RC + LC + IDC + OP + Ac + PO + C	2.68 (*p* = .000)	[0.040, 90% (0.038; 0.042)]	0.047	0.92	0.96	0.95	0.94

*IC* instrumental climate, *CC*  caring climate, *RC* rules climate, *LC*  law and code climate, *IDC* independence climate, *AC* affective organizational commitment, *PO* Person-organization fit, *OP* organizational pride; *C* organizational cynicism.

**Table 3:** The results of regression weights and average variance extracted (*N* = 1071).

Item	Factor	RW	AVE
What is best for everyone in the company is the major consideration here.	Caring climate	0.68	55.9
The most important concern is the good of all the people in the company as a whole.		0.70	
Our major concern is always what is best for the other person.		0.70	
In this company. people look out for each other’s good.		0.77	
People are expected to comply with the law and professional standards over and above other considerations.	Law and code climate	0.67	56.6
In this company. the law or ethical code of their profession is the major consideration.		0.75	
In this company. people are expected to strictly follow legal or professional standards.		0.83	
In this company. the first consideration is whether a decision violates any law.		0.75	
It is very important to follow the company’s rules and procedures here.	Rules climate	0.61	55.3
Everyone is expected to stick by company rules and procedures.		0.63	
Successful people in this company go by the book.		0.84	
People in this company strictly obey the company policies.		0.86	
In this company. people protect their own interests above all else.	Instrumental climate	0.58	44.8
In this company. people are mostly out for themselves.		0.69	
There is no room for one’s own personal morals or ethics in this company.		0.72	
People are expected to do anything to further the company’s interests. regardless of the consequences.		0.68	
In this company. people are expected to follow their own personal and moral beliefs.	Independence climate	0.78	57.6
Each person in this company decides for themselves what is right and wrong.		0.72	
The most important concern in this company is each person’s own sense of right and wrong.		0.82	
In this company. people are guided by their own personal ethics.		0.71	
The things that I value in life are very similar to the things that my organisation values.	Person-organization fit	0.78	78.6
My personal values match my organisation’s values and culture.		0.95	
My organisation’s values and culture provide a good fit with the things that I value in life.		0.92	
I feel happy for being a member of this company.	Organizationalpride	0.89	69.4
I feel happy to be an unforgettable part of this company.		0.88	
I am proud of the company’s achievements.		0.85	
This company offers something useful to the community.		0.70	
I am proud of the work I have done for this company.		0.67	
I am proud of my contribution to the success of this company.		0.72	
I feel proud when I tell others about my company.		0.85	
I do not feel ‘emotionally attached’ to this organization.	Affective commitment	0.76	62.9
This organization has a great deal of personal meaning for me.		0.92	
I do not feel a strong sense of belonging to this organization.		0.88	
I do not feel like ‘part of my family’ at this organization.		0.75	
I believe that my company says one thing and does another.	Organizational cynicism	0.82	64.4
My company’s policies, goals, and practices seem to have little in common.		0.83	
When my organization says it’s going to do something, I wonder if it will really happen.		0.70	
My company expects one thing of its employees, but rewards another.		0.78	
I see little similarity between what my organization says it will do and what it actually does.		0.90	
When I think about my organization, I experience aggravation		0.81	
1 feel that my company lacks integrity.		0.83	
I am not happy with the job at my company.		0.75	
My company irritates me.		0.77	

*RW* regression weights, *AVE* average variance extracted, *CR* composite reliability

In line with hypothesis 1, all types of ethical climates except for instrumental climate were positively related to person–organization fit, organizational pride, and affective commitment. The perception of an instrumental climate was negatively connected with person–organization fit, organizational pride, and affective commitment (Table 4). Hypothesis 2 was fully supported, as person–organization fit, organizational pride, and affective commitment were negatively associated with organizational cynicism.

**Table 4:** The results of correlation coefficients (*N* = 1071).

	1	2	3	4	5	6	7	8
Caring climate								
Law and code climate	0.35**							
Rules climate	0.38**	0.70**						
Instrumental climate	– 0.35**	− 0.06	− 0.15**					
Independence climate	0.48**	0.16**	0.14**	− 0.20**				
Person-organization fit	0.59**	0.28**	0.31**	− 0.40**	0.44**			
Organizational pride	0.55**	0.28**	0.30**	− 0.31**	0.31**	0.63**		
Affective organizational commitment	0.51**	0.18**	0.20**	− 0.37**	0.35**	0.58**	0.64**	
Organizational cynicism	− 0.56**	− 0.24**	− 0.32**	0.52**	− 0.35**	− 0.58**	− 0.64**	− 0.57**

#### Results: person–organization fit, organizational pride, and affective commitment as mediators

The hypothesis regarding the partially mediating role of person–organization fit, organizational pride, and affective commitment in the relationship between ethical climate and organizational cynicism was mostly supported. Both direct and indirect positive effects of the instrumental ethical climate on organizational cynicism were observed through person–organization fit, organizational pride, and affective commitment (Tables 5 and 6). Further, a caring climate was directly and indirectly negatively related to organizational cynicism through person–organization fit, organizational pride, and affective commitment. Meanwhile, law and code and independence climates were only indirectly negatively related to organizational cynicism through person–organization fit and organizational pride. A relevant, indirect effect was found in the relationship between a law and code climate and organizational cynicism through affective commitment, which was not a mediator between the independence climate, rules climate, and organizational cynicism. A rules climate was a negative predictor of organizational cynicism and was additionally related to it indirectly through organizational pride but not through person–organization fit.

**Table 5:** The results of standardized direct effects (*N* = 1071).

Outcome variable	Predictor variable	Estimate	se	CL 95%
Person-organization fit	Instrumental climate	− 0.18	0.02	[− 0.22, − 0.13]
Person-organization fit	Caring climate	0.35	0.03	[0.29, 39]
Person-organization fit	Rules climate	0.06	0.03	[-0.01, 11]
Person-organization fit	Law and code climate	0.06	0.03	[0.01, 11]
Person-organization fit	Independence climate	0.17	0.02	[0.12, 22]
Organizational pride	Instrumental climate	− 0.23	0.05	[− 0.33, − 0.13]
Organizational pride	Caring climate	0.61	0.06	[0.49, 0.71]
Organizational pride	Rules climate	0.16	0.07	[0.02, 0.28]
Organizational pride	Law and code climate	0.15	0.06	[0.02, 26]
Organizational pride	Independence climate	0.34	0.05	[0.23, 44]
Affective commitment	Instrumental climate	− 0.26	0.03	[− 0.32, − 0.19]
Affective commitment	Caring climate	0.38	0.04	[0.31, 46]
Affective commitment	Rules climate	− 0.01	0.04	[− 0.10, 07]
Affective commitment	Law and code climate	0.06	0.04	[− 0.02, 14]
Affective commitment	Independence climate	0.12	0.04	[0.05, 19]
Organizational cynicism	Person-organization Fit	− 0.33	0.07	[− 0.48, − 0.17]
Organizational cynicism	Organizational pride	-38	0.09	[− 0.57, − 0.20]
Organizational cynicism	Affective commitment	− 0.45	0.04	[− 0.54, − 0.36]
Organizational cynicism	Instrumental climate	0.72	0.06	[0.85, 26]
Organizational cynicism	Caring climate	− 0.33	0.08	[− 0.17, − 0.12]
Organizational cynicism	Rules climate	− 0.24	0.08	[− 0.08, − 0.09]
Organizational cynicism	Law and code climate	0.12	0.08	[− 0.02, 0.27]
Organizational cynicism	Independence climate	0.01	0.07	[− 0.13, 0.13]

**Table 6:** The results of standardized indirect effects (*N* = 1071).

Outcome variable	Predictor variable	Estimate	se	CL 95%
Total indirect effect				
Organizational cynicism	Instrumental climate	0.24	0.04	[0.16, 32]
Organizational cynicism	Caring climate	− 0.51	0.06	[− 0.63, − 0.40]
Organizational cynicism	Rules climate	− 0.09	0.05	[− 0.18, − 0.01]
Organizational cynicism	Law and code climate	− 0.10	0.04	[− 0.19, − 0.01]
Organizational cynicism	Independence climate	− 0.25	0.04	[− 0.34, − 0.17]
Partial indirect effect via person-organization fit				
Organizational cynicism	Instrumental climate	0.07	0.02	[0.03, 11]
Organizational cynicism	Caring climate	− 0.13	0.04	[− 0.21, − 0.06]
Organizational cynicism	Rules climate	− 0.02	0.01	[− 0.05, 0.00]
Organizational cynicism	Law and code climate	− 0.02	0.01	[− 0.05, − 0.01]
Organizational cynicism	Independence climate	− 0.07	0.02	[− 0.11, − 0.02]
Partial indirect effect via organizational pride				
Organizational cynicism	Instrumental climate	0.10	0.03	[0.04, 16]
Organizational cynicism	Caring climate	− 0.27	0.05	[− 0.36, − 0.19]
Organizational cynicism	Rules climate	− 0.07	0.03	[− 0.13, − 0.01]
Organizational cynicism	Law and code climate	− 0.07	0.03	[− 0.12, − 0.01]
Organizational cynicism	Independence climate	− 0.15	0.03	[− 0.22, − 0.09]
Partial indirect effect via affective commitment				
Organizational cynicism	Instrumental climate	0.06	0.02	[0.02, 12]
Organizational cynicism	Caring climate	− 0.10	0.03	[− 0.17, − 0.04]
Organizational cynicism	Rules climate	− 0.01	0.01	[− 0.02, 03]
Organizational cynicism	Law and code climate	− 0.02	0.01	[− 0.04, 0.01]
Organizational cynicism	Independence climate	− 0.03	0.01	[− 0.06, − 0.01]

The control variables that were irrelevant to organizational cynicism were sex (b = 0.82, SE = 0.54, 95% CI [–1.88, 0.22]), age (b = –0.01, SE = 0.01, 95% CI [–0.01, 0.01]), education (b = 0.29, SE = 0.48, 95% CI [–0.64, 1.23]), seniority (b = 0.03, SE = 0.09, 95% CI [–0.14, 0.21]), and company size (b = 0.37, SE = 0.20, 95% CI [–0.02, 0.76]). The only control variable that statistically significantly predicted organizational cynicism was the level of the position in the organizational structure (b = –0.80, SE = 0.20, 95% CI [–1.20, –0.39]).

To summarize, organizational pride was the only mediator of the link between all types of ethical climate and organizational cynicism. Person–organization fit and affective commitment did not mediate the relationship between a rules climate and organizational cynicism, and additionally an affective commitment did not mediate the relationship between a law and code climate and organizational cynicism.

## Discussion

This study was the first attempt to examine how employees’ perception of the ethical climate can reduce cynical attitudes in the workplace through affective organizational attachment, pride in the company, and perceived similarities in values between employees and the organization. Based on the results, some ethical climates, such as caring, instrumental, and rules climates, are not only indirectly but also directly related to organizational cynicism, which confirms previous findings regarding the role of ethical standards as an antecedent of employees’ approach to the organization^24–27^. It is important to explain why the law and code climate and independence climate were not predictors of cynical behaviors at work. This may have been related to the external ethical value systems underlying the implemented ethical rules, such as laws in the case of a code and law climate and the internal moral axiology of employees in an independence climate^9^. These ethical standards may not be as strongly identified with the organization as caring or instrumental climates, making it difficult for employees to identify with them, and they do not directly result in a cynical attitude.

From the employee perspective, focusing on the well-being of the highest number of organizational members in a caring climate or referring to dedicated ethical norms in a rules climate prevent cynicism in the workplace. Conversely, egoistic motives in the cues flowing from the perception of an instrumental climate may elicit cynical attitudes, which are grounded in selfishness^59^.

Besides the direct effects of ethical climates on organizational cynicism, organizational pride was the only mediator involved in the indirect relationship between all types of ethical climates and cynical attitudes. This implies that following social identity theory^30^, every ethical organizational climate besides an instrumental climate offers employees a way to identify with the company as attractive, admirable, desirable, a target to model, and something to be proud of. In turn, experiencing this beneficial emotional state helps to reduce cynicism.

It is also important to understand why an instrumental ethical climate negatively predicted organizational pride. It is possible that employees’ perception of an instrumental climate encourages them to pursue selfish needs, goals, and actions, which are not consistent with broader social goodness^42^. Thus, the values ​​that are promoted in an instrumental climate are viewed as unwanted and not as something to be proud of.

Consistent with social identity theory^32^, the results show that different types of ethical climates promote different ethical values and codes of conduct, which employees compare with their preferences to determine whether they identify with^30^. Similar to previous studies, similarities in the values and cultures of the company and employees were found to be beneficial, as suggested by person–organization fit theory^30^, protecting against organizational cynicism^4^. However, this effect was not observed in a rules climate. Further, the indirect effect of an instrumental climate on organizational cynicism through person–organization fit was positive, which shows that the selfish values ​​and attitudes imposed by instrumental ethical standards at work are contrary to those upheld by employees.

The last indirect mechanism between an ethical climate and organizational cynicism through effective commitment only manifested in caring, instrumental, and independent climates. This could be interpreted based on the reciprocity effect^60^ in social exchange^61^. A caring climate concentrates on the welfare of every employee and their needs. Employees perceive this as a manifestation of the organization’s interest in and concern for them, which elicits a desire to build an attachment to the company based on the rule of reciprocity as a type of debt payment and expression of gratitude, reducing organizational cynicism. Cicek et al.^5^ found that perceived organizational support was a negative predictor of organizational cynicism.

Similarly, receiving egoistic signals and perceiving a lack of concern from the company motivates reciprocity, which manifests as avoidance of organizational commitment and, consequently, the appearance of a cynical attitude in the workplace. These mechanisms did not occur in rules and law and code climates, likely because, in line with social exchange theory, it is difficult to assess favorable or unfavorable treatment from an organization when the ethical standards are based on universal law codes or are characteristic of the organizational ethical norms in politics and company procedures. Hence, they are not premises encouraging reciprocity.

This theoretical implications of this study relate to the beneficial role of person–organization fit, organizational pride, and affective commitment in the relationship between the organizational ethical climate and organizational cynicism. Except for an instrumental climate, all other types of ethical climates can reduce employees’ cynicism when they have an affective bond with the organization, perceive similarities with the organization, and are proud of the organization. Each of these aspects is based on the mechanism of social identification^32^ and is rooted in different needs. At the center of person–organization fit is the need for coherency between the organization and the employee. Organizational pride is grounded in the need for prestige as a chance to improve one’s status by identifying with the organization, which possesses desirable ethical attributes. Finally, affective commitment is related to the need for belongingness and the desire to develop a bond with the organization.

The practical implications of this study include the need to recognize the values and rules of the organizational ethical climate, excluding instrumental ones, as a way to foster employees’ identification with the organization to enhance person–organization fit, organizational pride, and affective commitment and protect against employee cynicism.

The Chief Executive Officers, Managing Directors, and Human Resources Managers should be conscious of the beneficial role of the organizational ethical climate, implement coherent, transparent, and predictable ethical standards, and effectively communicate them to employees as signposts they can follow in acting and making decisions. They should avoid building ethical standards on selfish premises to protect the companies against harmful effects, including employee cynicism.

Organizational values should be visible and transparent for every employee and have reflections on organizational and employee actions. Managers need to reward behaviors consistent with organizational values and show their subordinates the similarities between their values and the values of an organization, providing premises for organizational attachment and pride in the organization. Organizational actions within corporate social responsibility should be communicated, allowing employees to build organizational pride and identification with the organization. Thanks to that, the organizational representatives can ensure the positive attitude of employees toward the organization, avoiding organizational cynicism and harmful behaviors connected with it.

From the recruitment perspective, candidates should be verified for congruency between their preferred and organizational values, using not only methods based on declarations to avoid their cynical attitudes in the workplace. Companies that characterize an independent ethical climate need within the recruitment process to examine whether the ethical code of conduct of candidates to work, being the premise to make ethical decisions in the organization, is not focused on undesirable values connected with selfish, non-cooperative, and exploitative approaches to workmates.

The main limitation of this study is its cross-sectional design, which made it impossible to present the results from a cause-and-effect perspective, thus limiting them to indicating the probable direction between variables. Future longitudinal research could confirm the directions between the variables used in this model research and verify whether the organizational climate leads to person-organization fit, organizational pride, and commitment and, through these variables, influences organizational cynicism. An additional limitation regards the generalizability of the outcomes of this study. First of all, the first factor that influenced it was the non-random sampling method. Also, the lack of balance regarding sex distribution, with the vast majority of women, is an additional impactful element, which does not let the achieved results for all Polish employees be generalized.

## Conclusion

As was confirmed, ethical climates rooted in altruism and cooperation, employees’ ethical values and norms, or ethical rules based on a universal code of conduct like law or the Bible can prevent organizational cynicism thanks to building the affective attachment to the organization, pride in an organization, and values congruency between employee and company. On the opposite side, the instrumental ethical climate, which focuses on individual needs and well-being, and a self-centered approach, even at the expense of the workmates, makes it difficult to build affective bonds and pride in the organization, which can finally enhance a cynical attitude in the workplace.

Within practical implications, building ethical norms and rules in the organization on ethical values, opposite to selfishness and rivalry, regardless of the sources of the code of ethical conduct, is recommended. Longitudinal studies on the representative sample of Poles could confirm this study’s findings from the cause-and-effect perspective and increase the range of generalizability to all Polish employees.

## Data Availability

All data is available for the individual request directed to the author on correspondence address: marwnu@amu.edu.pl.
