# Author Correction: The relationship between ethical climate and organizational cynicism: mediating role of match and identification with the organization

**DOI:** 10.1038/s41598-025-28300-2

**Published:** 2025-11-18

**Authors:** Marcin Wnuk, Marta Żywiołek-Szeja, Agata Chudzicka-Czupała

**Affiliations:** 1 Department of Work and Organizational Psychology, Adam Mickiewicz University in Poznań, ul. Szamarzewskiego 89/AB, 60-568 Poznań, Poland; 2 Faculty of Psychology in Katowice, Interdisciplinary Center for Social Activity & Well-Being Research FEEL & ACT WELL, SWPS University, Katowice, Poland

Correction to: *Scientific Reports* 10.1038/s41598-025-97415-3, published online 09 April 2025

Marta Żywiołek-Szeja and Agata Chudzicka-Czupała were omitted from the author list in the original version of this Article.

The Author Contributions section now reads:

“Conceptualization, MW; data collection, ACC, MZS; data analysis, MW; writing original draft, MW; funding acquisition, ACC, MZS, MW; project administration, ACC, MW; final version acceptance, ACC, MZS, MW; correspondence, MW.”

The original Article has been corrected.

